# Conceptual foundations for a clarified meaning of the 3Rs principles in animal experimentation

**DOI:** 10.1017/awf.2024.39

**Published:** 2024-09-23

**Authors:** Edwin Louis-Maerten, Christian Rodriguez Perez, Rosa Maria Cajiga, Kirsten Persson, Bernice Simone Elger

**Affiliations:** 1 Institute for Biomedical Ethics, University of Basel, Basel, Switzerland; 2 Institute for Animal Hygiene, Animal Welfare, and Farm Animal Behaviour, University of Veterinary Medicine, Hannover, Germany; 3 Centre of Legal Medicine, Faculty of Medicine, University of Geneva, Geneva, Switzerland

**Keywords:** animal experimentation, animal research ethics, animal rights, animal welfare, policy, 3Rs

## Abstract

Russell and Burch’s 1959 original definitions of the 3Rs (replacement, reduction and refinement) are widely used today as standards for the ethical use of non-human animals in research, although they have a number of limitations. Authors and institutions around the world have addressed some of these, coming up in certain cases with more accurate, functional, and up-to-date definitions. However, not only do there still remain limitations needing to be addressed, but some that have been addressed resulted in discrepancies, contradictions, and general confusion as to how best apply the 3Rs in practice. In order to clarify the meaning of the 3Rs and enable more optimal implementation of these principles in animal experimentation, this article provides a theoretical discussion for revised definitions of the original 3Rs via examination of some of their main limitations and inconsistencies. First, we offer up the original definitions as presented in the context of Russell and Burch’s book *The Principles of Humane Experimental Technique.* Then, we examine the main limitations and present clear specifications and requirements for such revised definitions. After presenting our revised definitions, we conclude with various implications for animal welfare within the context of experimentation.

## Introduction

In 1959, Russell and Burch published their *Principles of Humane Experimental Technique*, in which they defined the concept of the 3Rs (Replace, Reduce, Refine). They gave specific definitions for each “R”:

“*Replacement means the substitution for conscious living higher animals of insentient material. Reduction means reduction in the numbers of animals used to obtain information of a given amount and precision. Refinement means any decrease in the incidence or severity of inhumane procedures applied to those animals which still have to be used*” [Russell & Burch 1992; chapter 4].

These three principles are now at the centre of many ethical discussions related to animal research. Even though many authors have suggested new directions for the ethical use of non-human animals in research (e.g. Aske & Waugh 2017; DeGrazia & Beauchamp 2019, 2020; Strech & Dirnagl 2019; Martin 2022; Brink & Lewis 2023; Bailey 2024), this article does not seek to explore alternatives to the 3Rs or complementary approaches. Rather, it will focus on *what the 3Rs are actually telling us*, i.e. on the very definition given to each of these principles, and how these definitions can relate to the current state of knowledge. Indeed, from their own acknowledgement, these definitions are “*broad*” (Russell & Burch 1992; chapter 4), “*clearly* [include] *areas of overlap*” (*ibid*; chapter 4), and sometimes integrate considerations that are not explicitly stated in them (e.g. the distinction between ‘relative’ and ‘absolute’ replacement). Since the concept of the 3Rs has become established during the previous decades, its increasing use as well as its broad understanding have given rise to various different interpretations in local laws, international regulations, or scientific publications. If we take the sole example of replacement, we can see how wide this scope of interpretation can be:

1. “*Replacement means the substitution for conscious living higher animals of insentient material*” [Russell & Burch 1992; chapter 4].
2. “*Member States shall ensure that, wherever possible, a scientifically satisfactory method or testing strategy, not entailing the use of live animals, shall be used instead of a procedure*” [EU Directive 2010/63 of the European Parliament and of the Council of 22 September 2010 on the protection of animals used for scientific purposes 2010; Art 4].
3. “*Replacement: Researchers should try to eliminate harm by replacing the animals targeted with entities that cannot be harmed [e.g. computer models, tissue cultures], or with animals of other species that would be harmed less*” [Curzer *et al*. 2016].
4. “*Replacement: Methods, which permit a given purpose to be achieved without conducting experiments or other scientific procedures on animals*” [Swiss 3RCC 2023].

In this context, unclear and inconsistent concepts hamper the implementation and acceptance of the 3Rs in animal research. An example of this inconsistency and need for clarification can be found when asking the question “*how should we account for the use of live animals currently considered as less sentient or insentient*?” e.g. many invertebrate species are still considered as insentient (Schukraft *et al.* 2019). If these live animals are considered completely insentient, then definitions (1) and (3) would deem this use as a suitable replacement technique, contrary to definitions (2) and (4). And, if these live animals are only considered as “*less sentient*” (although the idea of degrees of sentience may be disputed, see Schukraft *et al.* 2019), then only definition (3) would consider this use as a suitable replacement technique. How can we account for such a discrepancy? The debate at stake here is crucial since such a wide range of interpretation actually undermines the action of the 3Rs: if everybody has an intuitive interpretation of the 3Rs, they may well agree on the importance of implementing them but disagree on an actual plan of action due to the aforementioned discrepancies in interpretation. As Curzer *et al.* (2016) point out: “*we suspect that the broad appeal of the Russell and Burch’s Three Rs rests not only on their plausibility, but also on their vagueness. The ambiguity of the terms in which they are couched allows people with diverse views to endorse their own interpretations without deliberating with others upon their meaning or foundations.*” This may lead to conflicts with the original interpretation of Russell and Burch, e.g. the “*upturned hierarchy*” (Franco *et al.* 2018) that goes against the initial understanding of the British authors. More importantly, this may even lead to cases of “*whitewashing*” (Blattner 2019), i.e. cases where the 3Rs are deceptively marketed to convince the public that a private or public policy is compliant with an ethical approach to animal experimentation (Rodriguez Perez *et al.* 2023). Of course, Russell and Burch’s original definitions have been discussed over the years, and their lack of clarity has already been pointed out by various authors (Sandøe *et al.* 2015; Tannenbaum & Bennett 2015; Blattner 2019) but, as yet, no conceptual analysis of the 3Rs has been carried out, in order to clarify and comprehensively revise their meaning.

This article aims to provide just such an analysis by taking a fresh look at the original definition of Russell and Burch’s 3Rs and by providing a reasoned response to their main critics. To begin with, we will examine the original publication of Russell and Burch and seek to recover the implicit intentions they had in mind when first defining each “R.” Then, we will review the main revisions and limitations extolled in the literature and discuss their relevance for a clarified understanding of the 3Rs. A third part will be dedicated to our suggestion for a more consistent interpretation of the 3Rs. This is less to offer a definitive version of each “R”, with a wording that should remain unchanged, and more to provide a list of *desiderata* that each “R” must comply with in order to be fully functional and suitable for both conceptual research and concrete application in the field of animal experimentation. And, finally, we will discuss the relevance of such new definitions of the 3Rs and how clarifying them can also elucidate their role and scope in animal welfare.

## How did Russell & Burch originally define the 3Rs and for what purpose?

### *The context behind* The Principles of Humane Experimental Technique

As neatly summarised by Balls (2009a) and Kirk (2018), *The Principles of Humane Experimental Technique* (hereafter *Principles*) is a book requiring to be placed within a certain historical and epistemological framework. The *Principles* were commissioned in 1954 by the Universities Federation for Animal Welfare (UFAW), in particular major Charles Hume, with the objective of supplementing their *UFAW Handbook on the Care and Management of Laboratory and Other Research Animals* (for the latest edition, see Richardson & Golledge 2024) with an overview on humane techniques for animal research. At that time, the UFAW was treading a fine line. It did not want to pursue a political agenda as an antivivisectionist advocate, nor did it want to reduce the scientific validity of biological research but was genuinely concerned with the improvement of animal welfare and the reduction of suffering. To this end, they appointed William Russell, a zoologist, and Rex Burch, a biologist, to conduct field work and write the book, which was published in 1959. Russell and Burch followed a science-based approach to the definition and mitigation of suffering in laboratory animals. As they state themselves in the *Principles*:

“[T]*he words will be used in a purely objective sense to characterize the kind of treatment actually applied to an animal—in terms of the effect on the latter. Our use of the terms, henceforward, therefore, MUST NOT BE TAKEN TO IMPLY ETHICAL CRITICISM OR EVEN PSYCHOLOGIC DESCRIPTION OF PERSONS PRACTICING ANY GIVEN PROCEDURE”* [Russell & Burch 1992; chapter 2, accentuation in the original text].

Neither Russell nor Burch were moral philosophers; their view on harm resembles an arithmetical approach (i.e. suffering can be objectively measured and quantified, and some actions can either increase or decrease this amount), and their ensuing definition of the 3Rs was not intended to have moral value. This can lead to various sources of confusion when one wants to strictly apply the 3Rs as moral principles for animal research. Another possible source of confusion is the well-known challenging readability of the *Principles.* The complicated style of the book was mainly the work of Russell (Kirk 2018). The wording is often complex and the narrative lacks clarity, with many references to different disciplines intertwined, as emphasised by Hume himself:

“[T]*he style and presentation are really off-putting. The style is high-falutin’, complicated and obscure, and too long-winded. The references to psychoanalysis are of great interest to psychoanalysts, but hardly interesting to readers who have no knowledge of psychoanalysis, who will be in the majority. Many of the sentences have to be read more than once before the readers can construe them and see the point”* [Balls & Parascandola 2020].

Overall, the *Principles* are not a work out of context, and the understanding of this context can help to uncover the *actual* scope of action of the 3Rs. To paraphrase Balls, a dedicated reader of the *Principles* who published an abridged version (Balls 2009b):

“*Although a large number of people say they are committed to supporting the Three Rs concept of* Reduction, Refinement, and Replacement*, as put forward by Russell and Burch, most of them are unaware of the detailed implications of these insights and warnings, because they have not read the book itself. The result is that I am disappointed that the great benefits afforded by a careful consideration and dedicated application of* The Principles *have not been achieved”* [Balls 2010].

### The original objective of the 3Rs

The most critical concept of the *Principles* is not the 3Rs, but the more fundamental one of *inhumanity.* Indeed, the definition of the 3Rs first appears in the fourth chapter of the book, “*The sources, incidence, and removal of inhumanity*”, in a section called “*The Removal of Inhumanity: The Three R’s*”. According to Russell and Burch, the 3Rs (and the way they defined them) are essentially a means to remove inhumanity in experimental techniques, they ought not to be seen as an independent concept. We should first focus our attention therefore on the definition of inhumanity.

Russell and Burch dedicated an entire chapter to it. In the *Principles*, they defined humanity “*in a purely objective sense to characterize the kind of treatment actually applied to an animal*” (Russell & Burch 1992; chapter 2). Inhumanity therefore refers to a variety of negative mental states induced by the experimental technique and that animals experience, such as pain, fear, conflict, or frustration of need; these mental states being encompassed by the authors with “*the rather more general notion of distress*” (*ibid*; chapter 2). That is why “distress” and “inhumanity” are used interchangeably in the *Principles.* For clarity, we will only refer to “inhumanity” in this article. More precisely, the authors distinguish two kinds of inhumanity, direct and contingent inhumanity:

“*By the former, we mean the infliction of distress as an unavoidable consequence of the procedure employed, as such, even if it is conducted with perfect efficiency and completely freed of operations irrelevant to the object in view. By contingent inhumanity, on the other hand, we mean the infliction of distress as an incidental and inadvertent by-product of the use of the procedure, which is not necessary for its success*" [Russell & Burch 1992; chapter 4].

To illustrate these two kinds of inhumanity, painful necessary procedures are examples of direct inhumanity. Poor husbandry and stressful transportation, on the other hand, are examples of contingent inhumanity. According to the authors, contingent inhumanity can be easily mitigated by “*good husbandry practice, diligent care and common sense*” (Balls 2020), which is precisely the topic of the *UFAW Handbook on the Care and Management of Laboratory and Other Research Animals* (Richardson & Golledge 2024). Direct inhumanity, on the other hand, should be carefully considered when resorting to an experimental technique with a humanity criterion (Balls 2014):

“*If we are to use a criterion for choosing experiments to perform, the criterion of humanity is the best we could possibly invent. […] The greatest scientific experiments have always been the most humane and the most aesthetically attractive, conveying that sense of beauty and elegance which is the essence of science at its most successful*” [Russell & Burch 1992; chapter 8].

With these considerations in mind, we are now able to understand the actual objective of the 3Rs, as put forth by Russell and Burch: the diminution and, when possible, the removal, of *direct* inhumanity when using animals for experimental purposes, without compromising scientific and medical progress. In other words, the 3Rs are solely concerned with the inhumanity caused by the experimental technique (and by the experimental technique *only*), at an individual level for each animal, but also as a total sum of inhumanity for all animals being used in the experiment. And this concern ought to be balanced against those of scientifically sound research because they actually reinforce each other (Kirk 2018). With this in mind, we can already set two general *desiderata*, (G1) and (G2), for the definition of the 3Rs, according to Russell and Burch:

(G1) All 3Rs have the primary objective of reducing direct inhumanity in non-human animals used for experimentation.

(G2) All 3Rs are *pro tanto* principles, which means that they should be balanced against other principles, e.g. scientific value of the experiment.

### The original definition of the 3Rs

#### Replacement

The definition of a replacement method is stated as “*the substitution for conscious living higher animals of insentient material*” (Russell & Burch 1992; chapter 4). By substituting sentient beings with insentient materials, Russell and Burch argue that replacement aims at alleviating any kind of direct inhumanity imposed upon the research animals. The authors further divide replacement into two categories: absolute replacement, where sentient animals are not required at all at any stage, and relative replacement, where sentient animals are still required, but are exposed to no direct inhumanity (Russell & Burch 1992; chapter 5). Thus, the following *desiderata*, (Rep1) and (Rep2), are present:

(Rep1) Replacement is the complete alleviation of direct inhumanity.

(Rep2) Replacement should encompass both the possibility of absolute insentience (such material or living being could never experience direct inhumanity) and the possibility of relative insentience (the subject is somehow rendered insentient, e.g. by anaesthesia and analgesia, or the subject is sentient, but the experiment does not involve direct inhumanity, e.g. some designs of behavioural observations).

#### Reduction

The reduction principle is defined as the “*reduction in numbers of animals used to obtain information of a given amount of precision*” (Russell & Burch 1992; chapter 4). With this definition, Russell and Burch focus on the statistical design of the experiment. As Tannenbaum and Bennett (2015) pointed out, reduction is not about minimising or attempting to minimise the number of animals used, but about reaching a balance between ensuring enough statistical units to provide relevant scientific data, and not causing too many animals to suffer from direct inhumanity. This notion of “appropriate statistical power” is itself imprecise, since it relies upon the predictability of an experimental design, the statistical assumptions made on the experimental data, the statistical test used, or the level of significance chosen. Since these factors will vary according to the scientific field and the specific research hypothesis, it can be noted that there is a close proximity between a statistical power deemed “appropriate” and the scientific validity of a study. Therefore, the definition of reduction includes the following *desiderata*, (Red1) and (Red2):

(Red1) Reduction only refers to the statistical design of an experiment.

(Red2) Reduction aims at having just enough statistical units to reach appropriate statistical power in the experiment.

#### Refinement

Finally, the definition of refinement is stated as “*any decrease in the incidence or severity of inhumane procedures applied to those animals that still have to be used*” (Russell & Burch 1992; chapter 4). This is perhaps the most explicit principle, with a single *desideratum*, (Ref1). At the same time, as we shall see in the following section, the direct dependence upon the definition of direct inhumanity (or inhumanity in a broader sense) will be at the centre of many discussions.

(Ref1) Refinement is any means that can help to decrease direct inhumanity.

Now that we have made clear the original definitions of Russell and Burch, we have to consider and discuss their relevance. Limitations in Russell and Burch’s original 3Rs purpose and definition have been pointed out by numerous critics. For revised definitions of the 3Rs to be both functional and ethically suitable, they must take such limitations into account. Therefore, we will now discuss these main critics to be able to clarify the meaning of the 3Rs.

### Finding light in darkness: Main limitations and inconsistencies in the meaning of the 3Rs

The purpose of this section is to discuss and address the challenges that have been reported in relation to the 3Rs. Since it will not be possible to cover them exhaustively, we will focus purely on the limitations and inconsistencies that recur in the literature and that we consider the most problematic as regards attaining a clarified understanding of the 3Rs.

### Absence of ethical guidance

The most prominent critical aspect of the 3Rs in regards to their original definition is the absence of ethical guidance. We have seen that Russell and Burch’s work was commissioned at a time when welfarist initiatives for animals were increasing globally (e.g. the US Humane Slaughter Act enforced in 1958). However, most lacked a clear ethical framework and tended to bring together different views, such as utilitarian and right-based approaches, to facilitate more general progress in animal welfare (Francione 2018). In this sense, the 3Rs were not conceived as ethical principles and yet their main use today is to provide moral justification to animal experimentation, combined with other procedural requirements such as harm-benefit analyses. If we wish to use them as ethical principles, then we must define them more precisely within some normative framework, which should be coherent and justified. The choice of such a framework should be made explicit every time the 3Rs are discussed.

Arguing for a specific framework would be beyond the scope of this article. However, it can be noted that the current understanding of the 3Rs and animal experimentation in general is most often based on consequentialist approaches (Eggel & Camezind 2020). Other kinds of ethical frameworks may also be suitable to discuss the 3Rs, for instance principle-based approaches. In the context of research ethics, the 1978 Belmont report appears as a milestone with the principles of respect for the research subjects, beneficence and justice (Sims 2010). Three other approaches centred upon animal experimentation have been defended by DeGrazia and Beauchamp (2019, 2020), Martin (2022), and Brink and Lewis (2023). DeGrazia and Beauchamp (2019, 2020) suggest six principles based on the two core values of social benefit of animal research and the protection of animal welfare: (1) no alternative method, (2) expected net benefit, (3) sufficient value to justify harm, (4) no unnecessary harm, (5) basic needs, and (6) upper limit to harm. The authors consider these principles to be an appropriate framework for animal experimentation and “*close several gaps left by the Three Rs*” (DeGrazia & Beauchamp 2020; p 23). Alternatively, Martin (2022) argues that if one were to hold that animals have moral status and are therefore not merely tools for research, then the seven requirements for ethical research on humans proposed by Emanuel *et al.* (2000) may be applicable. These include: (1) social value, (2) scientific validity, (3) independent review, (4) fair subject selection, (5) favourable risk-benefit ratio, (6) informed consent, and (7) respect for research subjects. Finally, Brink and Lewis (2023) propose a more ‘conservative’ approach, as they build on the 3Rs, but produce a more comprehensive framework (the “12Rs framework”) by adding principles of social value (responsibility, respect, regulation), scientific integrity (relevance, reproducibility, transferability), and intersecting principles (reckoning, righteousness, reliability).

Again, we are not seeking to defend any particular one of these frameworks, but to emphasise that one is needed if we want the 3Rs to be an ethical concept for animal experimentation. Consequently, since the definition of the 3Rs has to be compatible with the specific framework within which they are embedded, there may be variations in some aspects of the definitions depending on the chosen framework (e.g. a utilitarian account of animal experimentation vs the use of DeGrazia and Beauchamp’s framework), leading to discrepancies in the understanding of the 3Rs. The requirement for an ethical framework can be captured by defining postulate (P1):

(P1) The proposed definition of the 3Rs is compatible with a well-defined ethical framework on the use of non-human animals in experimentation.

### Clarifying inhumanity with current knowledge on animal welfare

The second most pressing concern regarding the original definitions of the 3Rs is their reliance upon the notion of inhumanity. As shown above in *The original objective of the 3Rs*, according to Russell and Burch, inhumanity and distress may be considered interchangeably in the *Principles* and “*the words will be used in a purely objective sense to characterise the kind of treatment actually applied to an animal in terms of the effect on the latter*” (Russell & Burch 1992; chapter 2). Here, the issue in question is that, contrary to the 3Rs, the concept of inhumanity as understood by Russell and Burch no longer has any present day relevance as regards ethical discussion, legal texts, or scientific papers. How can one describe ‘inhumanity’ in modern terms? From our perspective, the term ‘inhumanity’ includes two dimensions: one of bad outcomes occurring to the laboratory animals (the “objective sense […] in terms of the effect on the latter”) and one of responsibility and disposition of the individual proceeding with the experimental technique (“the kind of treatment actually applied”). The first dimension is best captured with the notion of animal welfare, which uses physical and behavioural indicators to assess the well-being of the animals. The second extends beyond the concept of animal welfare and may be best characterised by the concept of respect for animal integrity, which calls for our responsibility to value the animals under our care. Thus, we argue that a modern way of defining the 3Rs should substitute the use of ‘inhumanity’ with “negative welfare or disregard to the integrity of animals.”

However, no such definitive and clear-cut definition of animal welfare (Mason & Mendl 1993; Fraser 2008; Mellor & Webster 2014; Mellor 2016) or animal integrity (Bovenkerk *et al.* 2002; Gavrell Ortiz 2004; Röcklinsberg *et al.* 2014) exists. Concerning animal welfare, it can be argued that, even in the absence of a definitive definition, we now know what constitutes positive and negative states of welfare, thanks to the development of welfare assessment methodologies such as the Five Freedoms model (Farm Animal Welfare Council 2009) or the more recent modified Five Domains model (Mellor *et al.* 2020). These assessment methodologies, although imperfect (see, e.g. Hampton *et al.* 2023), undergo continuous improvement through the progress being made in the field of animal welfare science, and this is exactly the kind of information that is needed in order for the 3Rs to be functional (i.e. to fulfil their objectives). On the other hand, including the notion of integrity enables us not only to focus on the state of welfare of the animal research subjects, but also to expand the consideration to the way scientists treat their research subjects. Following this, if the primary objective of the 3Rs is to reduce inhumanity, then the application of the 3Rs always implies some form of virtue ethics approach: reducing inhumanity involves a character trait or a disposition that is ‘more humane’ or ‘less inhumane.’ Some authors argue that the concept of animal dignity may be preferable to integrity in this case (see, e.g. Gavrell Ortiz 2004). However, due to the vagueness of its definition and the many debates regarding its usefulness as a concept (Macklin 2003; Loder 2016; Zuolo 2016; Bernet Kempers 2020; Shaw *et al.* 2024), we do not consider those aspects of dignity in this article. Therefore, here, from now on, the term ‘inhumanity’ will be replaced by “negative states of welfare or disregard for the integrity of animals.”

However, that leads us to another question: should the 3Rs refer only to the reduction of negative states of welfare and treatments that impinge upon the integrity of animals, or should they also consider the promotion of animal integrity or positive states of welfare? Current perspectives on animal welfare stress the importance of integrating positive states when making an assessment (Mellor & Beausoleil 2015). Similarly, the concept of animal integrity in positive law is gaining importance, e.g. in the Swiss Constitution (Art 120). The case of the Swiss constitution in this regard is peculiar, as it uses both the term ‘dignity’ in German (*Würde der Kreatur*) and Italian (*la dignità della creatura*), and ‘integrity’ in French (*l’intégrité des organismes vivants*). The term ‘dignity’ is retained in all languages in the Animal Welfare Act (Animal Welfare Act 2005; Art 3a). While Russell and Burch do not acknowledge the integrity of animals in the *Principles*, several times they stress the importance of the experimenter’s individual disposition towards the animals in the effort to improve experimental technique. And whether or not Russell and Burch wanted to include the promotion of positive states of welfare among the primary objectives of the 3Rs is a matter for debate (Tannenbaum & Bennett 2015). But it does seem clear from the *Principles* that promoting positive states of welfare is *at least* a way of reducing the amount of inhumanity:

“*It may be more satisfactory to think in terms of a scale than of two poles. In this way we are led to set our sights high in removing inhumanity, and to attempt always to drive the animal up to the highest possible point on the scale. Thus, we can aim at well-being rather than at mere absence of distress. Everything we know of the phenomena of suggestion is in favour of such a policy*” [Russell & Burch 1992; chapter 2].

Consequently, as noted by Tannenbaum and Bennett (2015), a more accurate interpretation of this quote would be “*that an effective way of diminishing and removing distress is sometimes to promote conditions in which animals are comfortable and experience wellbeing in some sense.*” Far from contradicting the primary objective of the 3Rs to reduce the occurrence of negative states of welfare, the inclusion of positive states of welfare can help to further achieve such reduction. Therefore, a logical location for these considerations would be within refinement, since they seem to detail the *desideratum* (Ref1) even more. Regarding the notion of integrity discussed above, it may be translated into a more general *desideratum* for the 3Rs, (G3):

(G3) All 3Rs relate to respect for animal integrity.

(Ref1) Refinement is any means, including the promotion of positive states of welfare, that can help to decrease the occurrence of negative states of welfare or disregard for animal integrity.

### Replacement as an absolute view against harm

A quite puzzling distinction that was made by Russell and Burch concerns the notions of absolute and relative replacement. As put forth in *desideratum* (Rep2), they defined absolute replacement in relation to absolute insentience, i.e. the experiment does not involve any harm being perpetrated to the experimental subjects because these subjects cannot be harmed (e.g. computational models, cell lines, insentient metazoan individuals). Similarly, they defined relative replacement in relation to relative insentience, i.e. the experimental subjects are sentient and therefore can be harmed, but the experimental procedure does not involve any harmful procedures, or such kinds of procedures are in some way nullified (Russell & Burch 1992; chapter 5). An example would be that of procedures performed under general anaesthesia and with suitable analgesia in which the individual is subjected to virtually no direct inhumanity. While we have no objection to the notion of absolute replacement, we argue that relative replacement should be abandoned because it merely (and mistakenly) refers to cases of refinement. Indeed, the mere use of experimental subjects that can be harmed makes it dubious, in practice, that there will be no harm involved whatsoever during the entire experimental technique (going back to the example of procedures performed under general anaesthesia, it is necessary to consider the harms involved before, during, and after that particular procedure, e.g. handling, housing adaptations, or potential medical complications, including the risk of dying). In this context, our opinion is that the best approach would be to acknowledge the potential harm inherent to the use of these subjects, and to consider any effort to mitigate it as a matter of refinement rather than replacement. This line of reasoning seems to be more in keeping with the notion that, e.g. general anaesthesia is being perceived as refinement rather than replacement (Buchanan-Smith *et al.* 2005; Flecknell & Thomas 2015). This clarification also resolves the conception that substituting individuals from a ‘higher’ place on the sociozoological scale with those from a ‘lower’ place on the scale, e.g. replacing a mammal with a fish or a sentient invertebrate (even though the mere existence of a sociozoological scale is criticisable from a biological perspective), ought not to be considered as a proper replacement method (Tannenbaum & Bennett 2015). As long as harm is involved, whether it is on a ‘lower’ or a ‘higher’ individual, one should not talk about replacement. Therefore, the *desideratum* (Rep2) should no longer be considered and *desideratum* (Rep1) may be amended, as follows, to only include the possibility of absolute insentience:

(Rep1) Replacement is the complete alleviation of negative states of welfare or disregard for animal integrity by the use of insentient material.

This notion of ‘insentient material’ has been recently investigated by Kramer (2024), who outlines further aspects to consider when evaluating whether a certain method qualifies as a true alternative. Following Kramer, a method is a suitable alternative to animal experimentation if it offers a reasonably effective and ethically acceptable response to the same scientific problem, under an appropriate description of that problem. Kramer further discusses whether an alternative should always be preferable to the standard method, but argues that in the case of animal experimentation, “*Even if animal experiments are preferable from an epistemic perspective, which is not evident for all domains of biomedical research, this does not exclude that animal-free approaches are acceptable or even preferable from a wider perspective*” (Kramer 2024). Therefore, setting aside the notion of preferability which needs further investigation, we can add the following *desideratum*, (Rep3), to replacement:

(Rep3) Replacement includes methods that must:

(Rep3-1) address the same problem as the considered animal experiment, under an appropriate description of that problem.

(Rep3-2) be sufficiently effective approaches to the research problem.

(Rep3-3) be ethically acceptable.

### Local vs general scope of application of the 3Rs

Another concern that needs clarification is the scope of application of the 3Rs: should they be limited to the sole experiment, or should they concern all aspects of a laboratory animal’s life or even animal experimentation in general? As we have seen in *The original objective of the 3Rs* with the distinction between direct and contingent inhumanity, according to Russell and Burch, there is no such thing as 3Rs for research in general or 3Rs for the animal’s whole life. The two authors were only referring to the reduction of *direct* inhumanity, the one that is *directly caused by the experimental technique*, when setting up the 3Rs. Since then, however, other authors have argued that the 3Rs, and specifically refinement, should also include *contingent* inhumanity, or negative states of welfare that are not imputable to the experimental technique (Buchanan-Smith *et al.* 2005; Richmond 2010).

We argue on the side of Russell and Burch here. Making the distinction between *direct* and *contingent* sources of negative states of welfare or disregard to animal integrity is precisely what brings the added value of the 3Rs: there are *specific* (*direct*) welfare concerns due to the experimental design or the context of research that yield *specific* ethical considerations, and the 3Rs are part of them, and there are *more general* (*contingent*) welfare concerns related to the fact that laboratory animals are *owned* animals and therefore should be protected by similar obligations from their owners as other kinds of owned animals (e.g. companion animals, farm animals, sport animals), and the 3Rs are not designed for this purpose. Two more reasons to keep such a local scope of application are that, first, the definition of the 3Rs would be clearer and, in theory, more straightforward to implement for scientists and policy-makers, since they would *only* need to focus on the scale of every single experimentation and their specific consequences on the welfare of the animals. Second, there is no risk of contradiction between the local and the general level, as some authors may suggest for the development of replacement methods (e.g. de Boo *et al.* 2005). Indeed, following de Boo *et al.* (2005), the main design to validate a replacement method (although epistemologically questionable) is the direct comparison with the conventional *in vivo* technique. If the 3Rs were to be interpreted in a general scope, there would have been conflict between replacement at the general scale (because the proposed new technique is supposed to be a suitable replacement method), and reduction at the local scale (because the validation study still has to use some animals for statistical analysis). This conflict (which also came under criticism from Eggel and Würbel 2021 for other reasons) does not hold any longer if one interprets the 3Rs at the level of the research project.

Therefore, we can modify the *desideratum* (G1) in order to take these considerations into account:

(G1) All 3Rs have the primary objective to reduce negative states of welfare or disregard for animal integrity that are directly attributable to an experiment.

One concern that could be raised with this discussion is that specific problems related to animal research, but not *directly* related to an experimental design, such as surplus animals, breeding practices, transportation, general husbandry, or genetic modifications to create specific phenotypes, are left out of the 3Rs. Some of them should indeed no longer be within the scope of the 3Rs (e.g. general husbandry, which is clearly stated by Russell and Burch as not being a matter of direct inhumanity, but “*a factor for contingent inhumanity in all types of experiments*” (Russell & Burch 1992; chapter 4), or transportation), but others are actually *directly caused* by the experimental design. For instance, if an experiment requires a very specific breed which, in turn, involves breeding many surplus individuals (individuals that will not be used for the experiment), it would be preferable to have less of these surplus individuals by either using another breed that does not have this downside or by improving the breeding scheme to maximise its efficiency. Another way to view this is to consider the breeding scheme for an experimental design as an experiment in itself, the objective of which is to produce a certain number of individuals with a particular genotype or phenotype. Concerning topics that are not directly related to an experimental design, far from being an issue, this consideration calls for more ethical and legal research, precisely because the 3Rs are not designed to tackle these problems. Co-operation should be sought at different levels (researchers, laboratory technicians, breeding facilities, animal caretakers, veterinarians, animal welfare specialists, policy-makers, ethicists, etc) in order to ensure that the welfare of research animals is also considered during their entire life (or more specific stages of their life), and not only during their experimental use. Guidelines such as PREPARE (Smith *et al.* 2018) may be used in this regard.

### Including death during the experiment as a harm

The next concern relates to the societal shift in how we consider animals, which indicates a common intuition that, in addition to causing them suffering, killing them (even painlessly) can also be considered as a harm. Whether death should be considered as a harm for an animal is a major cause for debate in the field of animal ethics (Kasperbauer & Sandøe 2016). However, many ethicists consider the painless killing of an animal which would otherwise have had a life of positive welfare as wrong, all things being equal (Carruthers 1992; McMahan 2002; Kagan 2016). This is true, for example, for classical utilitarianism and forms of preference utilitarianism (Carruthers 1992; Kagan 2016). The former considers killing in such a context as wrong because it prevents positive welfare (i.e. an objective value) from occurring, from an impersonal view. According to this, the harm is not done to the animal but is rather a harm from an impartial point of view. Some forms of the latter consider killing in such a context as a harm done to the animal, because the animal had (or would have had) a preference in experiencing that positive welfare. Moreover, if we consider deontological positions in animal ethics, it could also be argued that killing in such a context is a harm since it violates the right of the animal not to be treated instrumentally (Francione 1996). Here, we do not adopt a specific position, but simply agree with the conclusion that intentional killing that does not benefit the individual animal should be considered as a form of non-pathocentric harm, a disregard for their integrity, which is consistent with prominent positions in animal ethics, and also more in line with the current societal value in human-animal relationships.

As we have said, Russell and Burch were exclusively interested in the diminution and, if possible removal, of inhumanity. In that sense, their arithmetical approach in which harm is viewed as an objectively measurable property did not consider death as a harm. Considering our arguments above, it follows that the inability of the definition of the 3Rs to consider death as a harm is a major limitation. For that reason, we modify *desideratum* (G1) as follows:

(G1) All 3Rs have the primary objective to reduce negative states of welfare or disregard for the integrity of animals, including death, that are directly attributable to an experiment.

This modification enables different types of killings to be called into question. Of all animal deaths occurring in the field of animal research, at least two types are directly imputable to an experiment:

- Necessary killings for the experiment, i.e. animals killed to gather data.
- Necessary killings for mercy, i.e. animals killed because they reached an endpoint of unbearable suffering during the experiment.

While necessary killings for mercy may be seen as desirable from a welfare perspective because there is less harm than continued existence, necessary killing for the experiment is a type that should always be avoided. This can be translated into two new *desiderata*, (Rep4) and (Ref2):

(Rep4) Replacement does not include the killing of animals for the sole purpose of the experiment.

(Ref2) Refinement may include mercy killing when death is demonstrated to be a preferable option to continued existence.

Therefore, these considerations imply that an experimental design avoiding the killing of animals as part of the experiment is preferable to one which relies upon killings, all things being equal. This is also consistent with the growing issue of compassion fatigue and moral distress among professionals who have to kill research animals. Indeed, several studies have shown that the killing of research animals can impose significant psychological burdens that can diminish the well-being of laboratory animal personnel (Scotney *et al.* 2015; Newsome *et al.* 2019; King & Zohny 2022; Rumpel *et al.* 2023). Last but not least, this addition calls into question the status of research carried out on the body of animals killed in advance for that sole purpose. Such cases, like *in vitro* testing relying on previously killed animals, were considered as a form of relative replacement by Russell and Burch. That is, because the killing of these animals was not considered as a harm by the authors, who believed that the procedure was being conducted on insentient material (the body of a dead animal, or parts of it). Today, most regulations do not even consider these cases as animal research (e.g. EU Directive 2010/63 2010). However, since such deaths matter (as a non-pathocentric harm) and are directly imputable to the experiment, they should be included in the category ‘necessary killings for the experiment.’ Therefore, we argue that experimental methods relying on the killing of animals should not be considered as proper replacement, but rather as animal research *per se.* Of course, these methods continue to be greatly efficient in reducing the negative states of welfare imposed on research animals, but they should still be included within the scope of the 3Rs.

### Hierarchy and conflicts between the 3Rs

Many authors have discussed the conflicts that may arise between the 3Rs (e.g. de Boo *et al.* 2005; Olsson *et al.* 2012; Sandøe *et al.* 2015; Tannenbaum & Bennett 2015). It should therefore be clarified whether there is a strict hierarchy between these principles and how they should be defined in order to be mutually exclusive, that is in order to prevent considerations regarding one principle from contradicting considerations regarding another. From the reading of the *Principles*, it seems to be clear that replacement should always override the consideration of reduction or refinement: “*Replacement is always a satisfactory answer, but Reduction and Refinement should, whenever possible, be used in combination*” (Russell & Burch 1992; chapter 4), and these aspects should be included in their definition:

(G4) The 3Rs ought to be considered in a specific order:

(G4-1) If replacement is possible, then it must be done.

(G4-2) Reduction and refinement ought to be considered if, and only if, replacement is not possible.

However, whether there is some kind of hierarchy between reduction and refinement is less explicit. Russell and Burch emphasised how well-conducted refinement can help in the application of reduction (Russell & Burch 1992; chapter 8). Conversely, they were also aware that reducing too much can actually worsen the welfare state of animals (thus decreasing the effect of refinement). In this specific case, a balance between the individual level and the total welfare of the animals used in the experimental design needs to be reached. For instance, in an experiment originally involving drawing blood from 160 mice, one could suggest a scientifically equivalent design where only 40 mice are drawn four times. Such a design can be seen as a proper *minimisation* of the number of mice used, but if the welfare state of the 40 mice can be considered as worse, or reaching an unacceptable level, then it should not be considered as proper *reduction* (Tannenbaum & Bennett 2015). Another example would be the use of animals during the training of professionals: if no replacement method exists for the training of a given procedure, it may sometimes be preferable to prioritise refinement over reduction (e.g. by having one animal per trainee and using a low-stress method) or to prioritise reduction over refinement (e.g. by having a skilled trainer performing a high-stress method on only one individual in front of the trainees). Therefore, while acknowledging that no specific hierarchy exists between reduction and refinement (their order of application will depend upon each practical context of experimentation), a guiding *desideratum* for their definition would be that they should not *excessively* compromise with one another. Specifically, this means that refinement should not excessively compromise reduction, i.e. the number of statistical units used for the experiment, and that reduction should not excessively compromise refinement, i.e. the level of welfare compromise that each animal is subjected to. It can be argued that it is unclear at what point an increase becomes ‘excessive’, but this actually leaves room for a good informed judgment or *phronesis* to be made, e.g. in committees reviewing animal experimentation, as well as for potential changes in welfare science applied to the specific experimental design (e.g. a design may be intuitively considered as negatively impactful today, but demonstrated to not actually be so impactful some time later).

Therefore, three new *desiderata* can be defined, (G4-3), (Red3), and (Ref3):

(G4-3) Reduction and refinement do not have a hierarchical relationship.

(Red3) Reduction should not lead to an excessive increase in negative states of welfare or excessive disregard for animal integrity.

(Ref3) Refinement should not excessively increase the number of statistical units used for the experiment.

### Redefining and updating the 3Rs

The purpose of this section is to summarise the knowledge gained so far by providing a revised version of the 3Rs. To this end, Table 1 presents all postulates and *desiderata*, a suggestion of definitions deriving from these, and confronts these revised definitions with the original ones from Russell and Burch. Since the driving force of this article is clarity and precision, these revised definitions may appear overly sophisticated and, rather than facilitating better implementation of the 3Rs, may fail to be adopted. It is hoped that this article will serve as a conceptual base and lay some foundations to provide more workable definitions for laboratory animal scientists in the future.

**Table 1.** Summary of the revised definitions of the 3Rs

	Original definition	Desiderata	Revised definition
Postulate	N/A	The proposed definition of the 3Rs is compatible with a well–defined ethical framework on the use of non–human animals in experimentation.	N/A
General objective of the 3Rs	If we are to use a criterion for choosing experiments to perform, the criterion of humanity is the best we could possibly invent. […] The greatest scientific experiments have always been the most humane and the most aesthetically attractive, conveying that sense of beauty and elegance which is the essence of science at its most successful.	All 3Rs relate to the occurrence of negative states of welfare or disregard for the integrity of animals, including death, that are directly attributable to an experimentation*. All 3Rs have the primary objective of reducing this occurrence and promoting the respect for animal integrity. The 3Rs ought to be considered in a specific order: If replacement is possible, then it must be done. Reduction and refinement ought to be considered if, and only if, replacement is not possible. Reduction and refinement do not have a hierarchical relationship. All 3Rs are *pro tanto* principles, which means that they should be balanced against other principles, e.g. scientific validity of the experiment.	The 3Rs relate to the use of non–human animals for experimentation. More precisely, they refer to the negative states of welfare or disregard for the integrity of animals that are directly attributable to an experiment (hereafter “direct negative impacts”). The primary objective of the 3Rs is to reduce these direct negative impacts and to promote the respect for the integrity of those animals used for experimentation by following a strict hierarchy: replacement first, which should always be performed if available, and then reduction and refinement, which should be used in combination. Death imputable to the experiment should be considered as a direct negative impact and should also be mitigated. The 3Rs should be balanced against other considerations of the experiment, for instance its scientific validity.
Replacement	Replacement means the substitution for conscious living higher animals of insentient material. In relative replacement, animals are still required, though in the actual experiment they are exposed, probably or certainly, to no distress at all. In absolute replacement, animals are not required at all at any stage.	Replacement is the complete alleviation of direct negative impacts by the use of insentient material. Replacement includes methods that must offer a reasonably effective and ethically acceptable response to the same scientific problem, under an appropriate description of that problem. Replacement does not include the killing of animals for the sole purpose of the experiment.	Replacement is the complete alleviation of direct negative impacts. It can only be achieved by using materials or living beings that are insentient (e.g. *in vitro* models, *in silico* models, insentient animals, ethically sourced human or non–human cadavers). A method qualifies as a replacement if it is able to investigate a scientific problem in a reasonably effective and ethically acceptable manner. This excludes the possibility of killing the research subjects for the sole purpose of the experiment.
Reduction	Reduction means reduction in the numbers of animals used to obtain information of a given amount and precision.	Reduction only refers to the statistical design of an experiment. Reduction aims at having just enough statistical units to reach appropriate statistical power in the experiment^†^. Reduction should not lead to an excessive increase in direct negative impacts.	Reduction only refers to the statistical design of an experiment, by aiming at having just enough statistical units to reach appropriate statistical power in it. The application of reduction should not excessively increase the occurrence of direct negative impacts.
Refinement	Refinement means any decrease in the incidence or severity of inhumane procedures applied to those animals which still have to be used.	Refinement is any means, including the promotion of positive states of welfare, that can help to decrease the occurrence of direct negative impacts. Refinement includes mercy killing when death is demonstrated to be a preferable option to continued existence. Refinement should not excessively increase the number of statistical units used for the experiment.	In an effort to maintain the welfare of the research animals, refinement refers to any means, including the promotion of positive states of welfare, that can help to decrease the occurrence of direct negative impacts. Refinement includes the possibility of killing the research animals if death can be demonstrated to be a preferable option to continued existence. Moreover, refinement should not excessively increase the number of statistical units required for the experiment.

* For greater clarity in the following *desiderata*, we will use the terminology “direct negative impacts” in place of “negative states of welfare or disregard for the integrity of animals, including death, that are directly attributable to an experimentation”.

† See *Reduction* in *The original definition of the 3Rs* for details on what is considered as “appropriate statistical power”.

### Animal welfare implications

The 3Rs are widely accepted as a fundamental framework for conducting high-quality scientific experiments and developing alternative tools for enhancing animal welfare. Currently, the vast majority of countries and well-known international organisations, including the Council of Europe (Convention for the Protection of Vertebrate Animals Used for Experimental and Other Scientific Purposes, art 6[2], 7 and 8) and the World Organisation for Animal Health (Terrestrial Animal Health Code, art. 7.8.3), have adopted the 3Rs. In Europe, the 3Rs are now widely used as a point of reference by numerous committees and organisations whose mission it is to avoid animal experimentation to the greatest extent possible, or to improve conditions for laboratory animals. These principles are codified in many countries of the European Union through the directive 2010/63/EU on the protection of animals used for scientific purposes (Article 4), which calls for the application of the “*principle of replacement, reduction and refinement*” (Cozigou *et al.* 2015).

In addition, the 3Rs principles have been included in national and international regulations governing the use of animals in scientific procedures in order to introduce more humane experimental methods. While the broad adoption of the 3Rs principles has appeared to be a major success, these definitions and, therefore, its implementation may present many difficulties in protecting animals from negative human impact. In other words, even though the enshrinement of these principles in the current legislation appears to maximise the welfare of animals, there are claims that point out a regulatory failure (Blattner 2019). Continuing with the trend of previous years, Germany, France, Spain, Italy, Belgium, The Netherlands, Sweden, and Denmark have remained the EU member states with the highest numbers of animals used for scientific purposes. In total, more than 5.3 million animals were used in 2020 (European Commission 2020). However, a recent opinion poll in these eight member states highlighted the public’s desire to ultimately replace animals used for scientific purposes and accelerate the transition to non-animal science. (Eurogroup for Animals 2023).

Currently, the 3Rs remain a fundamental way to conceptualise animal research ethics. However, they were defined within a certain historical context by non-philosophers who based their thinking on a form of arithmetically based common morality that may give an initial impression of being perfectly satisfactory. This appearance led to the widespread implementation of the principles in the law and in laboratories, but more than half a century later, their vagueness is no longer acceptable for setting appropriate standards in animal experimentation. Since the 3Rs are implemented worldwide and have a great impact on the welfare of animal research subjects, their understanding should be universal and suitably clear for anyone using them, as well as in line with current societal values, ethical inputs, and a growing understanding of animal welfare. The objective of this article was to provide greater clarity and consistency as regards the understanding of the 3Rs, and to clearly demonstrate their actual strengths but also their weaknesses. With these revised definitions, the synergetic effect of the 3Rs is stressed with the general objective of safeguarding the welfare of animals at the level of the experiment. Each ‘R’ is defined with *desiderata* that are as precise as possible in order to explain and facilitate their implementation. Again, the article sought not to provide an exhaustive list, but to lay the groundwork for an understanding of the 3Rs that is both desirable and actionable for the relevant stakeholders, in particular those who actually make use of them in the field, such as researchers, animal care professionals, and animal experimentation committee members. In this context, it is critical to understand that having reliable definitions is not merely a matter of theoretical robustness, but also has significant consequences regarding the actual implementation in the law and the promotion of animal welfare in laboratories. As an example, the integration of these revised definitions in a 3Rs decision-making aid for professionals will allow up-to-date 3Rs decisions to promote both animal welfare as well as high-quality science, one of the many outcomes we hope will result from this article.

These revised definitions also create the opportunity to reconsider or perhaps downgrade the actual importance of the 3Rs in animal experimentation. Admittedly, the 3Rs have done a lot to increase awareness of animal welfare within this context, but they do not (and cannot) account for certain major aspects of animal research ethics. Some initiatives suggest complementary principles to the 3Rs (e.g. Eggel & Würbel 2021; Berliner Kompaktkurse 2023; Brink & Lewis 2023) while others simply abandon their use and propose valuable new frameworks (e.g. DeGrazia & Beauchamp 2019, 2020; Martin 2022). Another challenge is the current misuse of the 3Rs at the meta-level of animal experimentation (that is, not each single animal experiment, but the general way animal experimentation is envisioned and funded, for instance from a political perspective). Whether the objective is a complete phasing out of animal experimentation, a partial phase-out with emphasis on the protection of animals that still need to be experimented on, or something else, the actual application of the 3Rs by individual research teams would remain roughly the same, what would change would be the extent to which opportunities to apply them occurred (Eggel & Würbel 2021). In other words, the 3Rs cannot be a general objective *per se* for animal experimentation, because the extent of their implementation will depend upon the availability of non-animal methods and reduction and refinement techniques, which in turn depends on the research priorities set by the scientific community, public funding, private interests, and legal uptake (Müller 2023). In this context, more suitable frameworks would rely upon political theories (e.g. theories of change, see Müller 2022) in which the 3Rs are just one programmatic tool at the level of individual experiments.

## Conclusion

In conclusion, the current international guidelines for using animals in research seem to indicate that the 3Rs will remain the instrument of choice for policy-makers and administrators of animal facilities to use as a general compass to improve the welfare of animals used in research. In this article, our aim was to look at the definitions from Russell and Burch from the late 1950s and re-examine them under our current reality. Values in our societies are constantly evolving and adapting to new scientific discoveries. We therefore proposed several clarifications to the original meaning of the 3Rs to keep them aligned with the current stance on animal experimentation; we are convinced that solutions exist to harmonise the rising societal demands concerning the use of animals and the alleviation of animal suffering, and the push for scientific development to address the need of human and animal health. Since the 3Rs are now key concepts in animal experimentation, with the dual goal of conducting scientifically valuable research while respecting the animals involved and promoting their welfare, they deserve a clear interpretation that clarifies their potency and draws their limitations. There are at least two reasons for this: first, to facilitate the work of laboratory workers and related bodies (e.g. animal experimentation committees) who can have clear objectives to reach and, second, to fulfil our ethical duty towards the animals used for experimental purposes. The clarifications of the 3Rs we provide in this article may still be imperfect, but they open the door to more consistency in ethical, legal, and scientific debates on the matter.
